# The role of dental assessment in source identification during *Staphylococcus aureus* bacteremia: a scoping review

**DOI:** 10.3389/froh.2026.1876705

**Published:** 2026-07-17

**Authors:** Laura Isabell Werneburg, Jeremias Hey, Karl-Stefan Delank, Felix Werneburg

**Affiliations:** 1 Department of Prosthodontics and Geriatric Dentistry, Martin Luther University Halle-Wittenberg, Halle (Saale), Germany; 2 Department of Orthopedic and Trauma Surgery, Martin Luther University Halle-Wittenberg, Halle (Saale), Germany

**Keywords:** dental focus, infective endocarditis, oral infection, oral medicine, source control, *Staphylococcus aureus* bacteremia

## Abstract

**Objectives:**

*Staphylococcus aureus* bacteremia (SAB) is a severe bloodstream infection associated with substantial morbidity, mortality, and metastatic complications. Early source identification is essential for antimicrobial therapy, source control, treatment duration, and prevention of recurrence. However, the role of oral and dental sources in SAB remains insufficiently defined. This scoping review mapped clinical and microbiological evidence on oral and dental sources, oral *Staphylococcus aureus* reservoirs, and source attribution in SAB, sepsis, and infective endocarditis.

**Methods:**

A scoping review was conducted in accordance with the Population–Concept–Context framework and guided by PRISMA-ScR. PubMed/MEDLINE and Web of Science were searched for terms related to *Staphylococcus aureus*, bacteremia, bloodstream infection, sepsis, infective endocarditis, dental assessment, odontogenic infection, and oral colonization. Eligible sources included clinical studies, microbiological studies using human oral or dental samples, cohort studies, case reports, and case series addressing oral, dental, odontogenic, or orofacial relevance to *S. aureus*-related systemic infection. Data were charted according to study characteristics, oral/dental relevance, *S. aureus* relevance, contribution to source identification, and clinical implications.

**Results:**

Twenty-seven sources were included. Direct evidence mainly consisted of case reports describing severe MRSA or MSSA infections in which dental, oral, or orofacial foci were considered possible or probable sources. Endocarditis-related studies provided contextual evidence on dental procedures, oral screening, and dental status, but were rarely *S. aureus*-specific. Microbiological studies supported the oral cavity as a potential reservoir for *S. aureus*, MRSA, and resistant or virulent strains. Procedure-associated and high-risk host studies suggested possible links between oral procedures, mucosal disruption, odontogenic or oro-maxillofacial infection, and bacteremia or sepsis, although direct SAB source attribution remained limited.

**Conclusions:**

Current evidence does not support routine dental assessment for source identification in all patients with SAB. However, targeted dental evaluation may be clinically justified in selected situations, including persistent or unexplained bacteremia, suspected infective endocarditis, severe oral symptoms, odontogenic infection, salivary-gland infection, immunosuppression, or planned cardiac intervention. Future studies should use standardized dental assessment protocols and microbiological comparison of oral and bloodstream isolates to distinguish incidental oral pathology from true source attribution.

## Introduction

*Staphylococcus aureus* bacteremia (SAB) is defined by the detection of *Staphylococcus aureus* in blood cultures and should generally be regarded as clinically significant, as contamination is uncommon (1–3). SAB is one of the most clinically important bloodstream infections worldwide and represents a major challenge for clinicians across infectious disease, internal medicine, cardiology, surgery, intensive care, and dentistry (1, 3, 4). Its relevance is explained not only by the frequency of *S. aureus* as a cause of bloodstream infection, but also by its ability to cause persistent bacteremia, metastatic infection, and severe systemic complications (1, 3–8). Recent global burden analyses further underline the public-health relevance of *S. aureus*. In the Global Burden of Disease Study 2019, *S. aureus* was identified among the leading bacterial pathogens associated with mortality worldwide, and methicillin-resistant *S. aureus* was a major contributor to deaths attributable to antimicrobial resistance (9, 10).

The global burden of SAB remains substantial. Reported incidence rates range from approximately 20–50 cases per 100,000 population per year, and case fatality rates commonly range from 15% to 30% (1, 3, 6). In a pooled analysis of 3,395 adult patients from five prospective cohort studies, crude mortality was 14.6% at 14 days and 29.2% at 90 days (5). These data underline that SAB is not merely a microbiological finding, but a severe systemic infection associated with considerable morbidity, prolonged hospitalization, recurrence, and death (1, 3–6).

A major determinant of the clinical severity of SAB is its propensity for complicated and metastatic infection. Important complications include infective endocarditis, osteomyelitis, septic arthritis, vertebral osteomyelitis, spinal epidural abscess, psoas or splenic abscess, septic pulmonary emboli, infection of implanted material, and sepsis (3, 7, 8). Such complications may be clinically occult at the time of the first positive blood culture and can substantially influence treatment duration, need for imaging, source control, and prognosis (2, 3, 7, 8, 11–13). Therefore, SAB requires a structured diagnostic and therapeutic approach rather than isolated antimicrobial treatment (2–4, 11–13).

Early and systematic source identification is a central component of SAB management. Identification of the infectious focus guides antimicrobial selection, determines the required duration of therapy, enables timely source control, and supports risk stratification for persistent bacteremia, recurrence, metastatic infection, and mortality (2–4, 11–13). Contemporary SAB management therefore emphasizes repeated clinical assessment, follow-up blood cultures to document clearance, evaluation for infective endocarditis and metastatic infection, echocardiography when indicated, appropriate antimicrobial therapy, and prompt control or removal of infectious foci such as intravascular catheters, skin and soft tissue infections, osteoarticular infections, pneumonia, prosthetic material, and abscesses (2–4, 11–13). Infectious disease consultation has also been associated with improved quality of care and better short- and long-term outcomes in patients with SAB (14–16).

Despite structured diagnostic work-up, the source of SAB may remain unidentified in a subset of patients (3–5, 12, 13). In such cases, additional and less common potential foci require consideration. The oral cavity may represent one such site, because case-based evidence, microbiological studies, and procedure-associated or high-risk host studies support biologically plausible routes for bacteremia through dental, periodontal, mucosal, salivary-gland, and oro-maxillofacial conditions (17–36). Microbiological studies and recent background literature have shown that the oral cavity may harbor *S. aureus*, MRSA, resistant strains, and virulence-associated clones, supporting its potential relevance as a reservoir rather than solely as an incidental colonization site (24–27, 37–39).

However, the role of dental and oral infectious foci in routine SAB source identification remains insufficiently defined. Current SAB management recommendations emphasize source identification, source control, follow-up blood cultures, echocardiography, evaluation for metastatic infection, and interdisciplinary infectious disease management, but dental assessment is not clearly operationalized as a routine or conditional component of SAB work-up (2, 3, 11–13). In contrast, infective endocarditis guidelines, dental prevention statements, and cardiac-intervention-related dental literature address oral health mainly in the context of prevention, antibiotic prophylaxis for selected high-risk patients undergoing invasive dental procedures, regular dental care, and perioperative management before cardiac interventions (40–43). This creates an important gap between the recognized need for source identification in SAB and the unclear practical role of dental assessment when no source is apparent.

In clinical practice, dental assessment may be considered when no obvious source is identified, bacteremia persists despite appropriate antimicrobial therapy, oral symptoms or visible odontogenic infection are present, or infective endocarditis or cardiac intervention is under consideration. However, it remains uncertain whether oral findings meaningfully contribute to source identification or more often represent coincidental pathology. This distinction is clinically important because dental disease is common, whereas proven oral-source SAB appears to be rare and is difficult to confirm without microbiological linkage between oral and bloodstream isolates (17–28, 37–41).

This scoping review therefore examines the role of dental assessment and oral infectious focus evaluation in SAB from a clinical and interdisciplinary perspective. It aims to map available clinical and microbiological evidence on oral and dental sources, oral *Staphylococcus aureus* reservoirs, and source attribution in SAB, sepsis, and infective endocarditis. Because the evidence was expected to be heterogeneous and to include case reports, microbiological studies, endocarditis-related studies, and indirect clinical evidence, a scoping review design was chosen to map the breadth, type, and clinical relevance of available evidence rather than to estimate effect sizes or perform risk-of-bias-based synthesis.

## Methods

### Study design

This scoping review was conducted to map the available evidence on the role of oral, dental, odontogenic, and orofacial sources in the diagnostic work-up and source attribution of *Staphylococcus aureus* bacteremia (SAB), sepsis, and infective endocarditis. The scoping review includes clinical cohort studies, infective endocarditis studies, microbiological studies using human oral samples, case reports, and case series. The conduct and reporting of the review were guided by the PRISMA extension for Scoping Reviews (PRISMA-ScR). A completed PRISMA-ScR checklist is provided as Supplementary File 1, and the study selection process is summarized in a PRISMA flow diagram.

### Review question and PCC framework

The review question was: *What evidence exists on the role of oral and dental sources in the diagnostic work-up and source attribution of Staphylococcus aureus bacteremia and infective endocarditis?*

The eligibility criteria were structured according to the Population–Concept–Context framework.

#### Population

The population of interest included patients with *S. aureus* bacteremia, bloodstream infection, sepsis, or infective endocarditis, as well as patients in cardiac high-risk or cardiac intervention settings in which oral or dental infectious foci may be clinically relevant. Studies using human oral or dental samples were also considered eligible when they investigated oral *S. aureus* colonization or carriage as a potential reservoir related to transmission, infection risk, or systemic disease.

#### Concept

The concept of interest was the role of oral and dental sources in *S. aureus*-related systemic infection. This included dental assessment, dental consultation, oral or dental focus search, odontogenic infection, oral infectious foci, oral/maxillofacial source attribution, dental procedure-associated infection, oral source control, and oral *S. aureus* colonization or carriage when discussed as clinically relevant to reservoir potential, transmission, infection risk, or source attribution.

#### Context

The context was the diagnostic work-up and source attribution of *S. aureus* bacteremia and infective endocarditis. This included clinical settings involving source identification, infective endocarditis evaluation, interdisciplinary management, cardiac high-risk assessment, cardiac intervention planning, and oral reservoir assessment relevant to systemic *S. aureus* infection.

### Information sources and search strategy

A structured literature search was performed in PubMed/MEDLINE and Web of Science. The database searches were last updated in May 2026 and included records published up to that date. Search terms were developed to capture three major evidence domains: *S. aureus* bacteremia and oral/dental source attribution; oral *S. aureus* colonization and carriage; and dental assessment in infective endocarditis or cardiac high-risk settings.

The search combined terms related to *Staphylococcus aureus*, bacteremia, bloodstream infection, sepsis, infective endocarditis, dental assessment, odontogenic infection, oral infection, oral colonization, source identification, and source attribution. The PubMed/MEDLINE search included the following core terms:

“*Staphylococcus aureus*” OR “*S. aureus*”

AND

bacteremia OR bacteraemia OR “bloodstream infection” OR sepsis

AND

dental OR odontogenic OR “oral infection” OR “oral cavity” OR “dental focus” OR “oral focus” OR “source identification” OR “source attribution” OR “source control”

For Web of Science, the search strategy was broadened to include truncation and additional terms for infective endocarditis, dental screening, dental examination, dental consultation, and infectious focus. Reference lists of relevant full-text articles were also checked for additional eligible studies.

### Eligibility criteria

Sources were eligible for inclusion if they met at least one of the following criteria:

1. They reported original clinical or microbiological data relevant to *S. aureus*.
2. They addressed *S. aureus* bacteremia, bloodstream infection, sepsis, infective endocarditis, or metastatic *S. aureus* infection in relation to an oral, dental, odontogenic, salivary-gland, or orofacial source.
3. They assessed dental consultation, dental focus search, oral infectious foci, odontogenic infection, oral source control, or dental procedure-associated infection in the context of source identification or interdisciplinary management.
4. They examined oral, dental, salivary, mucosal, periodontal, denture-associated, or caries-associated *S. aureus* colonization or carriage in human samples when discussed as relevant to reservoir potential, transmission, systemic infection risk, or source attribution.
5. They investigated oral or dental infectious foci in patients with infective endocarditis, suspected infective endocarditis, heart valve disease, valve replacement, or cardiac intervention when findings were relevant to infection prevention, source attribution, or clinical management.
6. They were clinical cohort studies, cross-sectional studies, microbiological studies using human oral or dental samples, case-control studies, case reports, or case series.

Guidelines and high-quality reviews were not considered primary evidence sources for the scoping synthesis but were used for background, conceptual framing, and discussion of current clinical practice.

Sources were excluded if they:

1. addressed dental bacteremia or infective endocarditis without specific relevance to *S. aureus*;
2. addressed *S. aureus* infection, SAB, sepsis, or infective endocarditis without oral, dental, odontogenic, salivary-gland, or orofacial relevance;
3. investigated only nasal, skin, pharyngeal, or environmental *S. aureus* colonization without oral or dental sampling or clinical oral relevance;
4. focused on general oral health without relevance to *S. aureus*, bacteremia, sepsis, infective endocarditis, source attribution, or infection management;
5. were purely animal studies or laboratory studies without human oral/dental samples or clinical relevance;
6. were comments, editorials, letters, or narrative reviews without original data, unless used only for background discussion;
7. were conference abstracts or abstract supplements without sufficient extractable information;
8. lacked sufficient information to determine eligibility after title/abstract or full-text assessment;
9. described orofacial *S. aureus* infections clearly originating from cutaneous sources without relevance to the oral cavity, teeth, salivary glands, jaws, or odontogenic structures.

### Study selection

All records identified through PubMed/MEDLINE and Web of Science were screened for eligibility. After duplicate removal, titles and abstracts were screened against the predefined eligibility criteria. Full texts were retrieved for studies considered potentially relevant or unclear after title and abstract screening. Reasons for exclusion at the full-text stage were recorded using predefined categories, including no oral/dental relevance, wrong concept, no *S. aureus* relevance, no bacteremia/endocarditis/source relevance, review/editorial only, retracted article, duplicate, congress/abstract supplement, and insufficient information.

The screening and selection process was performed by two authors. Disagreements or uncertainties regarding eligibility were resolved through discussion until consensus was reached.

Because individual pre-consensus screening decisions were not retained in a format allowing retrospective calculation of Cohen's kappa, no formal inter-rater agreement statistic could be calculated.

References used only for background, guideline framing, quality-of-care context, and future research perspectives were not counted among the 27 included evidence sources. The included evidence sources are listed separately in the Results section.

### Data charting

Data from included sources were charted using a standardized extraction framework. Extracted variables included author, year, country, study design, population or setting, *S. aureus*/SAB relevance, oral or dental relevance, contribution to source identification, clinical consequences or outcomes, and overall relevance to the review question.

Because of the heterogeneity of the included evidence, sources were not pooled quantitatively. Instead, studies were organized into thematic evidence domains according to their primary contribution to the review question.

No review protocol was registered for this scoping review.

### Evidence synthesis

A narrative and thematic synthesis was performed. Included sources were grouped into four evidence domains:

1. direct clinical evidence for oral, dental, odontogenic, or orofacial sources of *S. aureus* systemic infection;
2. endocarditis-related and dental-screening evidence;
3. oral *S. aureus*/MRSA reservoir and microbiological evidence;
4. procedure-associated bacteremia and high-risk host evidence.

The synthesis focused on the extent to which the available evidence supports oral or dental source attribution, the distinction between biological plausibility and proven causality, and the potential clinical role of targeted dental assessment in selected patients with SAB or infective endocarditis. Particular attention was given to whether oral findings were described as incidental, possible, probable, or confirmed sources of infection, and whether microbiological linkage between oral and bloodstream isolates was available.

Relevance ratings were assigned narratively to indicate the contribution of each source to the review question. Sources were rated as high when they directly described *S. aureus* bacteremia, sepsis, metastatic infection, or infective endocarditis in relation to a clinically suspected oral, dental, odontogenic, salivary-gland, or orofacial focus. Sources were rated as moderate when they provided contextual evidence on dental assessment, oral infectious foci, oral *S. aureus* carriage, or high-risk settings without direct patient-level source attribution. Sources were rated as low to moderate when they supported biological plausibility or procedural relevance but did not address established SAB source identification.

## Results

### Study selection

**Figure 1:**
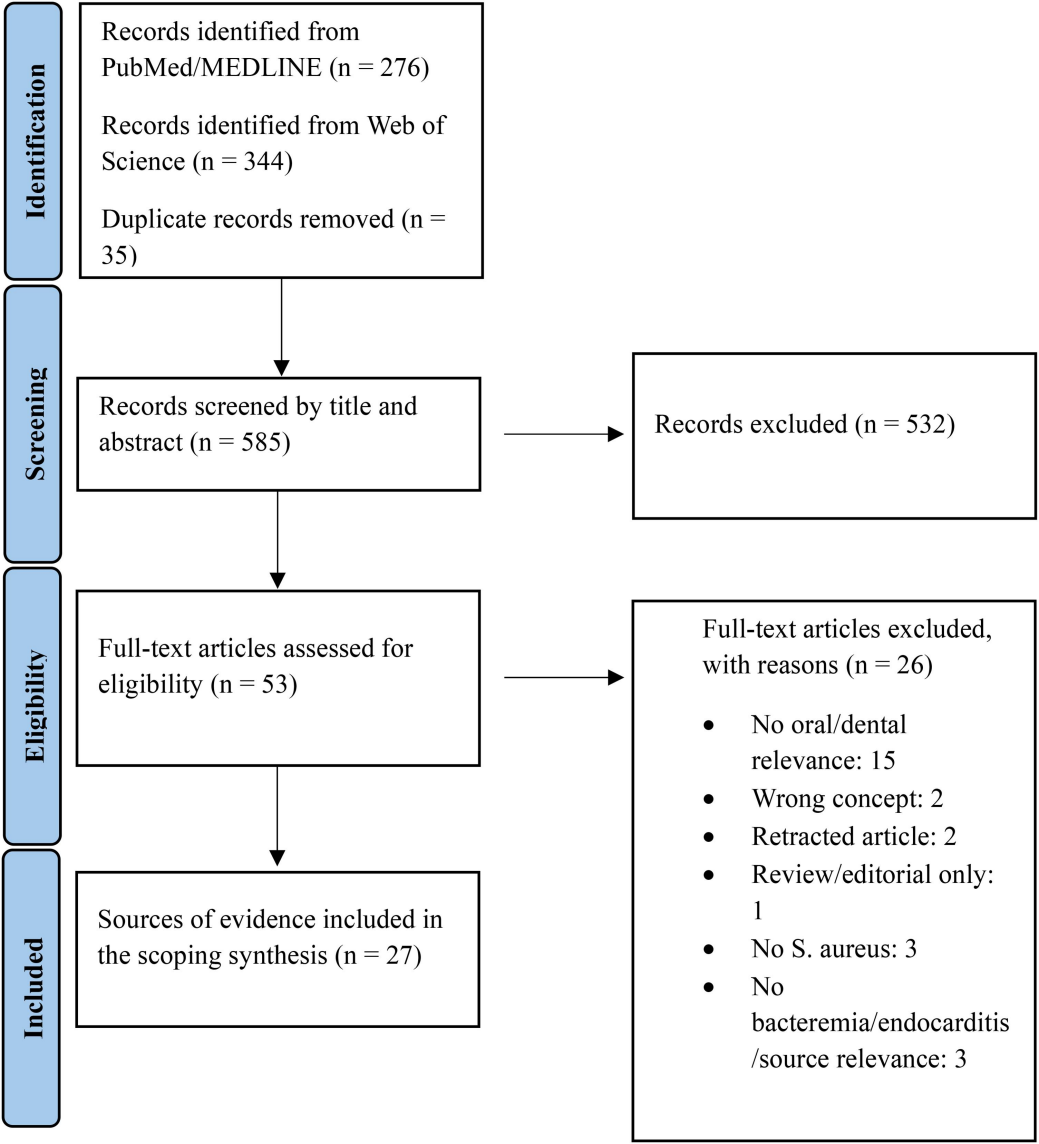
PRISMA-ScR flow diagram of the study selection process.

The study selection process is shown in Figure 1. Database searches identified 620 records, including 276 records from PubMed/MEDLINE and 344 records from Web of Science. After removal of 35 duplicates, 585 records underwent title and abstract screening. Fifty-three full-text articles were assessed for eligibility, and 27 sources of evidence were included in the scoping synthesis. Full-text articles were excluded because of lack of oral/dental relevance, wrong concept, retracted status, review/editorial-only publication type, no *S. aureus* relevance, or no bacteremia/endocarditis/source relevance.

### Characteristics of included evidence

The 27 included sources comprised case reports, cohort studies, registry-based or observational studies, microbiological studies using human oral or dental samples, and studies in high-risk or procedure-associated settings (17–36, 44–50). The evidence was heterogeneous and was grouped into four thematic domains: direct clinical evidence for oral, dental, odontogenic, or orofacial sources of *S. aureus* systemic infection (17–23, 28); endocarditis-related and dental-screening evidence (44–50); oral *S. aureus*/MRSA reservoir and microbiological evidence (24–27); and procedure-associated bacteremia or high-risk host evidence (29–36).

### Direct clinical evidence for oral, dental, odontogenic, or orofacial sources

The most direct evidence was derived from case reports in which oral, dental, odontogenic, or orofacial foci were considered possible or probable sources of *S. aureus* bacteremia, sepsis, toxic shock syndrome, metastatic infection, necrotizing fasciitis, or infective endocarditis (17–23, 28). Munoz et al. reported MRSA bacteremia with spinal infection in a patient with severe periodontal and endodontic disease, in whom hematogenous spread from the oral cavity was considered the most likely route of infection (17). Fardy et al. described fatal toxic shock syndrome in a child in whom the only identifiable infectious focus was an abscessed deciduous canine yielding TSST-1–producing *S. aureus* (18).

Similarly, Mikos et al. reported a fatal case of MSSA septicemia associated with retropharyngeal and epidural abscess formation, in which the odontogenic source was identified only after delayed dental consultation (19). Antunes et al. described cervical necrotizing fasciitis of odontogenic origin in a patient with uncontrolled diabetes, with MRSA isolated from culture and subsequent death from sepsis and multiorgan failure (20).

Other reports supported the broader relevance of orofacial sources. Basinger et al. described fatal MRSA bacteremia and toxic shock syndrome arising from acute parotitis in an immunocompetent adolescent, illustrating that non-dental but orofacial/salivary-gland sources may also be clinically relevant in *S. aureus* bloodstream infection (21). Blount and Leser reported multisystem complications after failed endodontic therapy and extraction, with MRSA-positive blood and operative cultures, although the precise origin of MRSA could not be definitively established (22).

Amedro et al. reported *S. aureus* infective endocarditis after ASD device closure in a child with untreated dental caries; however, the dental lesions were reported as a clinical context rather than a proven causal source (23). Na et al. described MRSA infective endocarditis in a 12-year-old girl who had undergone invasive dental treatment approximately 2 weeks before admission; although the dental procedure was discussed as a possible portal of entry, definitive causal attribution was not possible (28).

### Endocarditis-related and dental-screening evidence

Endocarditis-related studies provided contextual evidence on dental procedures, dental status, oral infection screening, and infective endocarditis microbiology (44–50). Thoresen et al. investigated 208 infective endocarditis patients who underwent oral infectious focus screening and found no statistically significant association between oral infection signs and infective endocarditis caused by viridans streptococci, suggesting that the diagnostic value of routine oral focus screening in infective endocarditis may be uncertain (44).

Ostovar et al. analyzed patients from the Brandenburg Endocarditis Registry and reported associations between recent dental treatment or desolate dental status and infective endocarditis, particularly with streptococcal organisms (45). However, these findings were less directly informative for *S. aureus*-specific source identification (45). Ismail et al. provided microbiological context by showing that staphylococcal infections were the most common infective endocarditis microorganisms in their cohort and that many infective endocarditis-associated organisms overlapped with taxa listed in the expanded Human Oral Microbiome Database (46).

Kumar et al. investigated pediatric infective endocarditis and found that *S. aureus* and viridans streptococci were among the major causative organisms; dental consultation was performed in a subset of patients during admission, supporting the clinical relevance of dental involvement in selected pediatric infective endocarditis cases, but without proving dental origin of *S. aureus* infection (50).

Additional epidemiologic studies supported the broader relevance of dental exposures in infective endocarditis (47–49). Delahaye et al. reported a dental portal of entry in 26.0% of infective endocarditis cases in a French regional survey (47). Loupa et al. identified *S. aureus* as the leading pathogen in a Greek infective endocarditis cohort and noted prior dental procedures in a subset of patients (48). Nakatani et al. similarly found that post-dental procedures were among the most common identifiable etiologic contexts of infective endocarditis in a nationwide Japanese survey, with MRSA reported in a minority of cases (49).

Overall, these studies support the contextual rationale for considering oral and dental factors in infective endocarditis and bloodstream infection workups (44–50). However, their contribution to *S. aureus*-specific dental source attribution remains limited and is primarily contextual rather than causal (44–46, 48–50).

### Oral *S. aureus*/MRSA reservoir and microbiological evidence

Several microbiological studies supported the plausibility of the oral cavity as a reservoir for *S. aureus* and MRSA (24–27). Smith et al. demonstrated that MRSA biofilms derived from oral and bloodstream isolates were not fully eradicated by commonly available mouthwashes, suggesting that oral MRSA biofilms may have infection-control relevance (24). Koukos et al. detected *S. aureus* in the oral environment of systemically healthy subjects, although MRSA was not identified and the authors considered oral *S. aureus* mainly as transient flora (25).

Kwapisz et al. provided particularly relevant molecular evidence, showing that both MSSA and MRSA major European clones could be isolated from the oral cavity of dental patients (26). The presence of virulence determinants, including enterotoxin genes and PVL-positive CA-MRSA strains, supports the interpretation that the oral cavity may serve as a reservoir for potentially invasive *S. aureus* strains (26). Similarly, Vellappally et al. reported MRSA and vancomycin-resistance markers among *S. aureus* isolates from dental caries specimens, highlighting the potential antimicrobial-resistance relevance of oral *S. aureus* colonization (27).

These studies do not demonstrate that dental assessment identifies the source of SAB (24–27). However, they strengthen the biological rationale for considering the oral cavity and dental disease as possible reservoirs, particularly in patients with poor oral health, immunosuppression, implanted cardiac material, or otherwise unexplained *S. aureus* bloodstream infection (24–27).

### Procedure-associated bacteremia and high-risk host evidence

A further group of studies addressed procedure-associated bacteremia and high-risk host settings (29–36). Adeyemo et al. showed that cleft lip and palate surgery was associated with polymicrobial bacteremia, including coagulase-positive *S. aureus*, although this represented transient postoperative bacteremia rather than clinically established SAB (29). Similarly, Akbulut et al. reported bacteremia after orthodontic debonding, with bacterial growth detected after removal of composite residues and plaque deposits; *S. aureus* was among the recovered organisms, supporting biological plausibility but not direct SAB source attribution (30).

Several studies focused on immunocompromised or otherwise high-risk populations (31, 32, 35, 36). Akintoye et al. investigated whether radiographic periodontal disease predicted septicemia after allogeneic HSCT and found no significant association between alveolar bone loss and septicemia of likely periodontal or oral origin, despite frequent positive blood cultures and recovery of organisms including *S. aureus* (31). Mawardi et al. provided similarly important tempering evidence in patients with multiple myeloma and MRONJ undergoing HCT: *S. aureus* bacteremia occurred in three patients, but no patient developed pain or infection at the MRONJ site during hospitalization (32).

Other studies supported the broader relevance of oral and oro-maxillofacial infection in systemic sepsis (33–36). Egwari et al. reported postsurgical sepsis after odontogenic tumor surgery, with polymicrobial infections including *S. aureus* and with antibiotic susceptibility testing guiding therapy (33). Constantinescu et al. examined antimicrobial resistance among pathogens implicated in sepsis with an oro-maxillofacial portal of entry and emphasized the therapeutic importance of *S. aureus* resistance patterns (34). Olczak-Kowalczyk et al. showed that damaged oral mucosa in immunocompromised children may be accompanied by bacteremia, while Peterson et al. suggested that acute periodontal infections during myelosuppression may represent a portal of entry for bacteremia (35, 36).

Overall, these sources strengthen the mechanistic rationale for considering oral and dental sites in selected patients with bacteremia, particularly those with recent oral procedures, impaired host defenses, mucosal barrier injury, or severe odontogenic/oro-maxillofacial infection (29–36). However, their contribution to the review should be framed as supportive and contextual, because they generally do not demonstrate that dental assessment directly identifies the source of established *S. aureus* bacteremia (29–36).

### Summary of evidence

Across the included evidence, oral, dental, odontogenic, and orofacial sources of *S. aureus* systemic infection were described mainly in case reports and selected high-risk contexts (17–23, 28–36).

Microbiological studies support the plausibility of the oral cavity as a reservoir for *S. aureus*, MRSA, and resistant or virulent strains (24–27). Endocarditis and high-risk host studies provide additional contextual evidence but rarely establish patient-level causal attribution (31, 32, 35, 36, 44–50). Therefore, the available evidence supports selective rather than routine dental assessment in SAB, with the strongest rationale in patients with unexplained or persistent bacteremia, suspected infective endocarditis, severe oral symptoms, odontogenic infection, salivary-gland infection, immunosuppression, or planned cardiac intervention.

**Figure 2:**
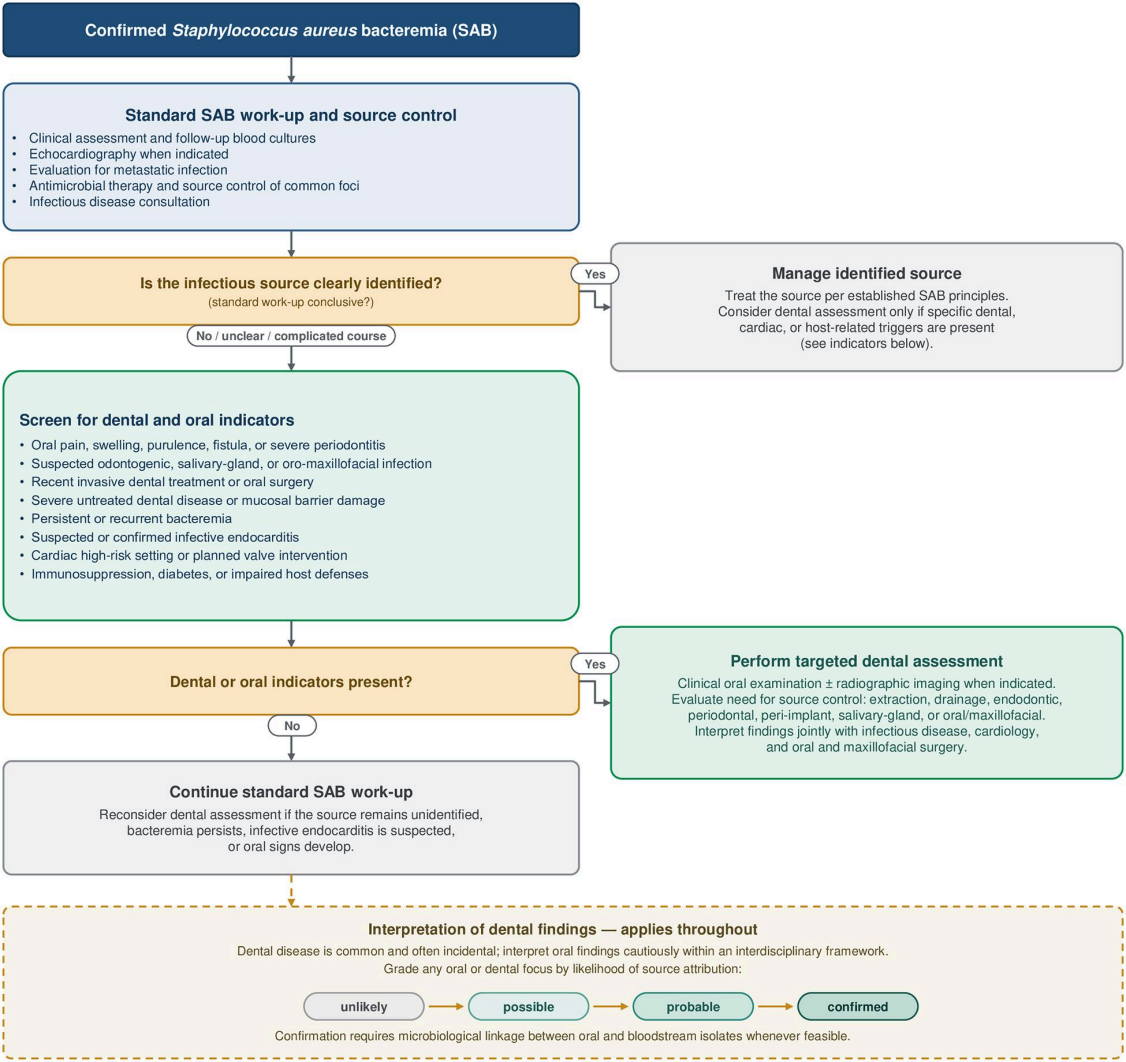
Risk-based approach to considering dental assessment in *Staphylococcus aureus* bacteremia.

Based on the mapped evidence, targeted dental assessment appears most clinically relevant in selected SAB patients rather than as a routine diagnostic step in all cases. Figure 2 summarizes a suggested decision flow for considering dental assessment in relation to source identification, complicated clinical course, oral findings, infective endocarditis, cardiac risk, and host-related risk factors.

## Discussion

### Principal findings

This scoping review mapped the available evidence on the role of oral, dental, odontogenic, and orofacial sources in source identification and clinical attribution during *Staphylococcus aureus* bacteremia, sepsis, and infective endocarditis. Overall, the evidence suggests that oral and dental sources of *S. aureus* systemic infection are biologically plausible and clinically relevant in selected cases, but they are not established as frequent source categories in unselected SAB populations.

The strongest direct evidence was derived from case reports in which dental, oral, or orofacial foci were considered possible or probable sources of MRSA or MSSA bacteremia, sepsis, toxic shock syndrome, spinal infection, necrotizing fasciitis, or infective endocarditis (17–23, 28). These reports demonstrate that severe oral or odontogenic disease can occasionally be associated with systemic *S. aureus* infection. However, the case-based nature of this evidence limits conclusions regarding frequency, predictive value, and the diagnostic yield of routine dental assessment in all patients with SAB.

Endocarditis-related studies provided additional context by showing that dental procedures, dental status, and oral infection screening are commonly considered in infective endocarditis workups (44–50). However, these data are often more strongly linked to viridans streptococci than to *S. aureus*. Studies on oral *S. aureus* colonization and microbiological reservoir potential support the biological plausibility that the oral cavity may harbor MSSA, MRSA, resistant strains, and virulence-associated clones (24–27). Procedure-associated and high-risk host studies further suggest that oral procedures, mucosal barrier disruption, periodontal infection, medication-related osteonecrosis of the jaw, and oro-maxillofacial infections may be associated with bacteremia or sepsis, particularly in vulnerable hosts (29–36). Nevertheless, these studies generally do not establish patient-level causal attribution between oral findings and SAB.

### Biological plausibility versus proven source attribution

The oral cavity represents a complex microbial ecosystem and may function as a reservoir for *S. aureus* under selected conditions. Microbiological studies included in this review detected *S. aureus* or MRSA in oral samples, including plaque, tongue, caries-associated specimens, and oral biofilms (24–27). Molecular evidence further indicates that oral *S. aureus* isolates may carry antimicrobial resistance determinants and virulence factors, supporting their potential clinical relevance beyond simple colonization (26, 27).

However, biological plausibility must be clearly distinguished from proven source attribution. The presence of *S. aureus* in the oral cavity does not necessarily mean that the oral cavity is the portal of entry for bacteremia. Oral colonization may coexist with nasal, skin, catheter-related, or other sources of *S. aureus*. Similarly, dental disease is common in many patient groups affected by SAB, including older adults, medically compromised patients, and those with poor access to oral health care. Therefore, the detection of dental pathology during SAB workup may represent incidental comorbidity rather than the origin of bloodstream infection.

This distinction is particularly important because most included studies lacked microbiological linkage between oral and bloodstream isolates. Without molecular comparison, it remains difficult to determine whether an oral strain and a bloodstream isolate are clonally related. Even in reports where dental or orofacial infection was clinically plausible, definitive proof of causality was rarely available (17–23, 28). Thus, oral findings should be interpreted along a graded probability spectrum—unlikely, possible, probable, or confirmed source—rather than being automatically classified as causal.

### Dental assessment in relation to current SAB and endocarditis guidance

Current recommendations for SAB management emphasize early source identification, source control, follow-up blood cultures, evaluation for infective endocarditis and metastatic infection, appropriate antimicrobial therapy, and infectious disease consultation (2, 3, 11–13, 16). These principles are directly relevant to the present review, because the potential value of dental assessment depends on whether oral or dental findings can meaningfully contribute to source identification or source control. However, dental assessment is not clearly operationalized as a routine or conditional component of standard SAB work-up in current management recommendations (2, 3, 11–13). This is also consistent with general clinical management reviews of SAB, which emphasize prompt recognition of clinically significant bacteremia, repeat blood cultures, echocardiographic evaluation when indicated, assessment for metastatic infection, appropriate antimicrobial therapy, infectious disease involvement, and source control, but do not define dental assessment as a standard diagnostic step (11).

Infective endocarditis guidelines, dental prevention statements, and cardiac-intervention-related dental literature address the oral cavity mainly in the context of prevention, oral hygiene, antibiotic prophylaxis for selected high-risk patients undergoing invasive dental procedures, and dental management before selected cardiac interventions (40–43). They do not specifically define when dental assessment should be performed for source identification in patients with established SAB. This gap is clinically relevant because SAB management requires timely identification and control of the infectious focus, while oral findings are common and may be incidental. The findings of this review therefore support a selective and risk-based approach rather than routine dental assessment in all SAB patients.

### Clinical interpretation of dental findings in SAB

The findings of this review do not support routine dental assessment for all patients with SAB. Established SAB management already prioritizes identification and control of common sources, including intravascular catheters, skin and soft tissue infection, osteoarticular infection, pneumonia, prosthetic devices, abscesses, and infective endocarditis (3–5).

In this context, the absence of odontogenic infection as a frequent source category in large SAB cohorts suggests that routine dental screening in unselected SAB populations would likely have limited yield (5).

However, the review also indicates that dental assessment may be clinically valuable in selected situations. The strongest rationale exists when bacteremia remains unexplained after standard diagnostic evaluation, persists despite appropriate antimicrobial therapy, or occurs together with oral symptoms or visible odontogenic infection. Clinical red flags include acute dental pain, facial or intraoral swelling, purulence, fistulation, severe periodontal inflammation, peri-implant infection, mucosal breakdown, salivary-gland infection, or severe untreated dental disease (17–23, 28).

Dental evaluation may also be relevant in patients with suspected or confirmed infective endocarditis, particularly when cardiac surgery, valve intervention, or prosthetic material is involved (23, 28, 40, 44–50). In such cases, dental assessment may serve more than one purpose: it may contribute to source attribution, identify active oral foci requiring treatment, support perioperative planning, and reduce the risk of future bacteremia from untreated oral disease. Nevertheless, the evidence reviewed here suggests that dental findings in infective endocarditis should still be interpreted cautiously, especially when the causative organism is *S. aureus* rather than a typical oral streptococcus (44–50).

### Patient groups in whom dental assessment may be most relevant

The included evidence suggests several clinical contexts in which targeted dental assessment may be particularly justified. First, patients with persistent or unexplained SAB may benefit from dental evaluation when standard source investigations do not identify a plausible focus (3–5). Second, patients with SAB and suspected infective endocarditis, prosthetic valves, intracardiac devices, or planned valve intervention may represent a group in which oral findings have practical implications for both infection management and procedural planning (23, 28, 40, 44–50).

Third, immunocompromised or otherwise high-risk patients may warrant closer attention to oral sources. Studies in hematopoietic stem cell transplantation, myelosuppressed cancer patients, and children with secondary immunodeficiency suggest that oral mucosal damage, periodontal infection, and jaw pathology may be associated with bacteremia or systemic infection in vulnerable hosts (31, 32, 35, 36). Fourth, patients with severe odontogenic or oro-maxillofacial infection, especially in the setting of diabetes or impaired host defenses, may be at increased risk for deep-space infection, sepsis, and metastatic complications (19, 20, 22, 33, 34).

Dental assessment may therefore be best conceptualized as a selective adjunct rather than a universal diagnostic step. Its value is likely highest when oral findings are acute, symptomatic, purulent, or anatomically connected to spreading infection, and lowest when findings are chronic, asymptomatic, or nonspecific.

### Implications for interdisciplinary management

The findings of this review underscore the importance of communication between infectious disease specialists, cardiologists, dentists, oral and maxillofacial surgeons, and other treating clinicians. Dental evaluation in SAB should not merely document the presence of caries, periodontal disease, or radiographic abnormalities. Rather, it should provide a clinically meaningful interpretation of whether the oral findings are likely to be incidental or potentially relevant to the bloodstream infection.

A structured dental report in SAB or infective endocarditis could include: the presence or absence of acute odontogenic infection; signs of purulence, swelling, fistulation, or deep-space spread; periodontal or peri-implant inflammation; mucosal breakdown; salivary-gland infection; radiographic evidence of acute or chronic dental foci; and an overall judgment regarding source likelihood. Because causal attribution is often uncertain, terminology such as “unlikely,” “possible,” “probable,” or “confirmed” oral source may be more appropriate than categorical statements of causality.

When an oral or dental focus is considered clinically relevant, source control should be coordinated with the overall infectious disease treatment plan. This may include extraction, drainage, debridement, periodontal or endodontic intervention, or management of salivary-gland infection. However, the risks of dental intervention must be balanced against the patient's systemic condition, anticoagulation status, cardiac risk, need for urgent surgery, and timing of antimicrobial therapy.

### Methodological limitations of the evidence base

The evidence base identified in this review has several important limitations. First, direct evidence was largely restricted to case reports and case-based observations (17–23, 28). Although these reports demonstrate that oral or odontogenic *S. aureus* systemic infection can occur, they cannot estimate prevalence, diagnostic yield, or comparative effectiveness of dental assessment.

Second, many included studies were not designed to evaluate dental source attribution in SAB. Endocarditis studies often focused on oral streptococci, dental procedures, or oral screening more broadly, whereas *S. aureus*-specific conclusions were limited (44–50). Similarly, microbiological studies demonstrated oral *S. aureus* carriage or antimicrobial resistance but did not establish bloodstream invasion or source causality (24–27).

Third, uncommon sources are often poorly characterized in large SAB cohorts. Dental or odontogenic foci may be hidden within residual categories such as “other,” “unknown,” or “miscellaneous,” limiting the ability to determine their true frequency (5). Conversely, studies from dental or cardiac settings may overestimate the relevance of oral findings because they include populations already selected for oral assessment or cardiac risk.

Fourth, there is a lack of standardized definitions for what constitutes a clinically relevant dental focus in SAB. Acute abscess, purulence, and spreading infection are likely to have a different meaning than chronic apical radiolucency, stable periodontitis, or asymptomatic caries. Yet current studies rarely provide enough detail to distinguish these entities in a reproducible way.

Finally, microbiological confirmation is usually missing. Few studies compare oral isolates with blood culture isolates using molecular typing or whole-genome sequencing. This absence of strain-level linkage is a major barrier to distinguishing true oral-source SAB from coincidental oral colonization or dental disease.

### Strengths and limitations

A strength of this scoping review is that it integrates several evidence domains that are usually considered separately: SAB source attribution, oral *S. aureus* colonization, infective endocarditis and dental screening, case reports of odontogenic or orofacial systemic infection, and high-risk host studies. This broad approach is appropriate for a topic in which the evidence is heterogeneous and direct data are sparse.

However, the review also has limitations. The included sources differed substantially in design, setting, population, and outcome definitions, precluding quantitative synthesis. The evidence was mapped thematically rather than pooled statistically. In addition, because many included studies were not primarily designed to evaluate dental source identification in SAB, conclusions must remain cautious.

The search was limited to PubMed/MEDLINE and Web of Science. Although these databases were considered appropriate for capturing biomedical, infectious disease, cardiology, and interdisciplinary dental literature relevant to SAB, infective endocarditis, and oral/dental evidence, Embase and the Cochrane Library were not searched. Therefore, relevant records indexed exclusively in these databases may have been missed. In addition, because the available evidence was heterogeneous and often indirect, the review can identify clinically plausible indications for targeted dental assessment but cannot determine the diagnostic yield, sensitivity, specificity, or cost-effectiveness of dental evaluation in SAB.

Furthermore, although screening was performed by two authors and disagreements were resolved by consensus, individual pre-consensus screening decisions were not retained in a format allowing retrospective calculation of Cohen's kappa. Therefore, no formal inter-rater agreement statistic could be reported.

## Future directions

Future research should move beyond simply detecting oral pathology in patients with SAB and instead focus on causal attribution and clinical utility. A first priority is prospective study designs in unselected or clearly defined SAB populations using standardized dental assessment protocols, including structured clinical examination, reproducible radiographic criteria, and predefined categories for classifying oral findings according to their likelihood of representing an active infectious focus (17, 44).

A second major priority is improved microbiological linkage between oral and bloodstream isolates. Current studies on the oral cavity and *S. aureus* primarily address carriage, colonization, and antimicrobial resistance, but rarely determine whether oral strains are genetically related to isolates recovered from blood cultures (37–39). Future investigations should therefore combine oral sampling with molecular comparison of isolates, ideally using high-resolution genomic techniques, in order to distinguish mere coexistence from true transmission or hematogenous spread (37, 38). Existing molecular studies have already shown that oral *S. aureus* strains may carry clinically relevant resistance and virulence profiles, but comparable analyses linking oral and bloodstream isolates in SAB are still lacking (26, 38).

Another important direction is the development of clinically applicable risk stratification tools. At present, dental consultation in SAB is largely guided by general clinical judgment, for example in the setting of persistent bacteremia, unknown source, suspected infective endocarditis, or visible oral disease (3, 5, 41). Future studies should identify which combinations of systemic and oral findings are most predictive of clinically meaningful dental involvement. This could support more efficient interdisciplinary decision-making and help avoid both underrecognition of relevant oral foci and overattribution of incidental dental pathology (3, 5, 44).

Research should also evaluate whether and how dental assessment affects management in specific high-risk groups. Patients with suspected infective endocarditis, planned valve intervention, prosthetic material, or substantial comorbidity may represent subgroups in whom oral findings have greater practical relevance for source control, perioperative planning, or prevention strategies (40, 41). Prospective outcome-oriented studies in such populations would be particularly valuable.

Finally, future work should place greater emphasis on clinically meaningful endpoints beyond simple detection of oral abnormalities. Relevant outcomes include time to source identification, modification of management, need for dental intervention, microbiological clearance, recurrence of bacteremia, and mortality (3, 5). This would help determine whether dental assessment in SAB has value not merely as an additional diagnostic exercise, but as an intervention that can improve care in selected patients.

## Conclusion

Current evidence does not support routine dental source investigation in all patients with *Staphylococcus aureus* bacteremia. Oral, dental, odontogenic, and orofacial sources are biologically plausible and have been described in selected cases, but direct evidence remains limited and largely case-based (17–23, 28). Microbiological studies support the oral cavity as a potential reservoir for *S. aureus*, MRSA, and resistant or virulent strains, but do not establish dental source attribution in SAB (24–27).

Targeted dental assessment appears most appropriate in selected clinical situations, including persistent or unexplained bacteremia, suspected infective endocarditis, severe oral symptoms, odontogenic infection, salivary-gland infection, immunosuppression, or planned cardiac intervention.

Because oral pathology is common and microbiological confirmation is usually lacking, dental findings should be interpreted cautiously and within an interdisciplinary framework. Prospective studies with standardized dental assessment and molecular comparison of oral and bloodstream isolates are needed to clarify when dental evaluation meaningfully contributes to source identification, source control, and patient outcomes in SAB.
