# Supplementary material for: The role of dental assessment in source identification during Staphylococcus aureus bacteremia: a scoping review
Source: Front Oral Health. 2026 Jul 17;7:1876705. doi: 10.3389/froh.2026.1876705 (PMC13423864; doi:10.3389/froh.2026.1876705)
Supplement: Supplementary file 1 [file Supplementaryfile1.docx]

**Supplementary File 1. PRISMA-ScR Checklist**

**Reporting guideline:** Preferred Reporting Items for Systematic reviews and Meta-Analyses extension for Scoping Reviews (PRISMA-ScR). Checklist item wording is based on Tricco et al. (2018). Page and line numbers refer to the manuscript version dated 22 June 2026.

| **Section** | **Item** | **PRISMA-ScR reporting item** | **Reported on page/line** | **Notes** |
| --- | --- | --- | --- | --- |
| TITLE | 1 | Identify the report as a scoping review. | p. 1, lines 1-3 | Reported in the manuscript title. |
| ABSTRACT | 2 | Provide a structured summary including background, objectives, eligibility criteria, evidence sources, charting methods, results, and conclusions. | p. 2, lines 30-59 | Structured abstract reports objectives, methods, results, conclusions, and keywords. |
| INTRODUCTION | 3 | Describe the rationale for the review in the context of what is already known and why a scoping review approach is appropriate. | p. 3-4, lines 66-125 | Rationale and need for evidence mapping are described in the Introduction. |
| INTRODUCTION | 4 | Provide an explicit statement of the questions and objectives, referring to key elements such as population, concept, and context. | p. 4-5, lines 119-152 | Objective and review question are stated; PCC framework is reported. |
| METHODS | 5 | Indicate whether a review protocol exists and where it can be accessed, or state that no protocol was registered. | p. 7, line 225 | No review protocol was registered. |
| METHODS | 6 | Specify characteristics of sources of evidence used as eligibility criteria and provide the rationale for these criteria. | p. 5-6, lines 139-203 | Population, concept, context, inclusion criteria, and exclusion criteria are reported. |
| METHODS | 7 | Describe all information sources used and the date of the most recent search. | p. 5, lines 153-169 | PubMed/MEDLINE and Web of Science were searched; the last update was in May 2026. |
| METHODS | 8 | Present the electronic search strategy for at least one database, including limits used, so that it can be repeated. | p. 5, lines 154-169 | Core PubMed/MEDLINE terms and the Web of Science search approach are reported. |
| METHODS | 9 | State the process for selecting sources of evidence, including screening and eligibility assessment. | p. 6-7, lines 204-214 | Title/abstract screening, full-text assessment, duplicate removal, and consensus procedure are described. |
| METHODS | 10 | Describe the methods of data charting and any process for obtaining and confirming data from included sources. | p. 7, lines 218-224 | Standardized data charting framework and thematic organization are described. |
| METHODS | 11 | List and define all variables for which data were sought and any assumptions or simplifications made. | p. 7, lines 219-222 and 237-243 | Extracted variables and narrative relevance ratings are described. |
| METHODS | 12* | If done, provide the rationale and methods for critical appraisal of individual sources of evidence. | p. 7, lines 237-243; p. 16-17, lines 521-534 | No formal critical appraisal was performed; sources were narratively rated for relevance and limitations are acknowledged. |
| METHODS | 13 | Describe the methods of handling and summarizing charted data. | p. 7, lines 226-243 | Narrative and thematic synthesis across four evidence domains is described. |
| RESULTS | 14 | Give numbers of sources screened, assessed for eligibility, and included, with reasons for exclusions at each stage, ideally using a flow diagram. | p. 8-9, lines 244-270 | PRISMA-ScR flow diagram and exclusion reasons are reported. |
| RESULTS | 15 | Present characteristics of included sources of evidence. | p. 9, lines 271-277 | Study designs and four thematic evidence domains are summarized. |
| RESULTS | 16* | If done, present critical appraisal results for included sources of evidence. | Not applicable | No formal critical appraisal was conducted for this scoping review. |
| RESULTS | 17 | For each included source of evidence, present the relevant charted data related to the review questions and objectives. | p. 9-12, lines 278-381 | Results are reported by evidence domain and source type. |
| RESULTS | 18 | Summarize or synthesize the results of included sources of evidence. | p. 12, lines 369-381; p. 14-16, lines 384-515 | Summary of evidence and discussion distinguish biological plausibility from proven source attribution. |
| DISCUSSION | 19 | Summarize the main results and link them to the review questions and objectives. | p. 12, lines 369-381; p. 14, lines 384-405 | Principal findings and implications are summarized. |
| DISCUSSION | 20 | Discuss limitations of the scoping review process. | p. 16-17, lines 497-534 | Limitations include case-based evidence, database restrictions, heterogeneity, lack of standardized definitions, missing microbiological linkage, and missing kappa calculation. |
| DISCUSSION | 21 | Provide a general interpretation of the results with implications for future research and practice. | p. 17-18, lines 535-577 | Future research directions and conclusions are provided. |
| FUNDING | 22 | Describe sources of funding for the included sources and for the scoping review, and the role of funders. | p. 19, lines 595-608 | The review reports that no specific grant funding was received. |

**Items 12 and 16 are optional according to PRISMA-ScR.*
