# Supplementary material for: The role of dental assessment in source identification during Staphylococcus aureus bacteremia: a scoping review
Source: Front Oral Health. 2026 Jul 17;7:1876705. doi: 10.3389/froh.2026.1876705 (PMC13423864; doi:10.3389/froh.2026.1876705)
Supplement: Supplementary file 2 [file Table1.pdf]

Appendix

Extraction Table – Direct Clinical Evidence Sources

**Author/Year**	**Country**	**Study Design**	**Population/Setting**	***S. aureus*/SAB Relevance**	**Oral/Dental Relevance**	**Contribution to Source Identification**	**Clinical Consequences**	**Relevance**
**Munoz et al. 2023 [17]**	USA	Case report	54-year-old man with Crohn's disease, spinal infection, and MRSA bacteremia	Multiple blood cultures were positive for MRSA; the patient had paraspinal abscess, discitis, and arachnoiditis.	Poor dentition, erythematous gingiva, chronic dental pain, missing molars, and radiographic evidence of periodontal/endodontic disease.	The oral cavity was discussed as the likely source, with hematogenous spread from a dental infection to the bloodstream and subsequently to the spine.	Intravenous antimicrobial therapy; interdisciplinary evaluation; no evidence of infective endocarditis on transesophageal echocardiography.	**High**
**Fardy et al. 1999 [18]**	United Kingdom/Wales	Case report	9-year-old girl presenting with toxic shock syndrome, renal failure, and overwhelming septic shock	Pus from the dental abscess grew TSST-1–producing *Staphylococcus aureus*.	Periodontal abscess associated with an upper right deciduous canine.	The dental abscess was considered the only identifiable infectious focus; the authors emphasized oral surgical review in patients with toxic shock syndrome of unclear origin.	Tooth extraction, antimicrobial therapy, intensive circulatory and multiorgan support; fatal outcome despite treatment.	**High**
**Basinger et al. 2018 [21]**	USA	Case report / forensic case report	13-year-old immunocompetent adolescent with acute parotitis, toxic shock syndrome, sepsis, and death	Blood cultures obtained at admission and autopsy grew MRSA; MRSA parotitis was considered a rare cause of bacteremia in an immunocompetent adolescent.	Orofacial/salivary-gland focus; no evidence of poor dentition, but severe left-sided parotitis was present.	Parotitis was considered the primary source of MRSA bacteremia based on clinical presentation, disease progression, and autopsy findings.	Rapid deterioration with shock, disseminated intravascular coagulation, respiratory failure, acute renal failure, and death.	**Moderate to high**
**Mikos et al. 2025 [19]**	Poland	Case report	65-year-old man with retropharyngeal and epidural abscesses, sepsis, and fatal outcome	Blood cultures revealed methicillin-susceptible *S. aureus* (MSSA).	Extensive dental caries and poor oral hygiene identified during dental consultation.	The initial working diagnosis was urosepsis; dental involvement was suspected only during preoperative airway evaluation, leading to	Drainage of the retropharyngeal abscess, tracheostomy, antimicrobial adjustment, multiple dental extractions; death	**High**

Author/Year	Country	Study Design	Population/Setting	*S. aureus*/SAB Relevance	Oral/Dental Relevance	Contribution to Source Identification	Clinical Consequences	Relevance
						dental consultation. An odontogenic source was ultimately considered the most probable origin.	due to heart failure secondary to septicemia.	
**Blount and Leser 2012 [22]**	USA	Case report	54-year-old man with poorly controlled diabetes after failed endodontic therapy and extraction of a mandibular premolar	Blood cultures and operative cultures were positive for MRSA; acute bacterial endocarditis was suspected but not confirmed by transesophageal echocardiography.	Failed endodontic therapy, extraction, and subsequent deep odontogenic infection involving multiple fascial spaces.	The odontogenic infection was the apparent initiating event for severe systemic complications, although the precise route and origin of MRSA contamination could not be definitively established.	Incision and drainage, extraction, intravenous antibiotics including vancomycin, intensive care, ARDS, septic embolic pattern of cerebral infarcts, prolonged hospitalization.	**Moderate to high**
**Antunes et al. 2013 [20]**	Brazil	Case report	Patient with uncontrolled diabetes and extensive cervical necrotizing fasciitis of odontogenic origin	Culture was positive for MRSA; the patient died after sepsis and multiorgan failure.	Odontogenic infection as the origin of cervical necrotizing fasciitis.	Demonstrates that an odontogenic focus may progress to severe MRSA-associated cervicofacial infection with systemic deterioration.	Broad-spectrum antibiotics, surgical drainage and debridement; fatal outcome due to sepsis and organ failure.	**High**
**Amedro et al. 2017 [23]**	France	Case report and literature review	8-year-old boy with late infective endocarditis 3 years after transcatheter ASD device closure	Blood cultures grew oxacillin-susceptible *S. aureus*; the literature review identified *S. aureus* as the most frequent bacterium in reported cases of endocarditis after atrial defect device closure.	Several untreated dental caries were noted; dental care was performed during the clinical course.	Dental caries were documented as a relevant clinical context, but a causal relationship between the dental lesions and *S. aureus* endocarditis was not proven.	Gentamicin and oxacillin therapy, dental treatment, and surgical device removal; cerebral microemboli were detected on MRI.	**Moderate to high**
**Na et al. 2016 [28]**	South Korea	Case report	12-year-old girl initially presenting with presumed bacterial meningitis and later	Blood cultures grew MRSA; echocardiography revealed mitral valve vegetation; MRSA endocarditis was diagnosed.	The patient had undergone invasive dental treatment, including gingival treatment and braces placement,	The dental procedure was discussed as a possible portal of entry, although the source of MRSA acquisition	Prolonged intravenous antimicrobial therapy; cerebral embolic lesions/cerebritis;	**Moderate to high**

Author/Year	Country	Study Design	Population/Setting	*S. aureus*/SAB Relevance	Oral/Dental Relevance	Contribution to Source Identification	Clinical Consequences	Relevance
			diagnosed with MRSA infective endocarditis		approximately 2 weeks before admission.	could not be definitively proven.	recovery without valve surgery.	

Extraction Table – Indirect Clinical and Endocarditis-Related Evidence

Author/Year	Country	Study Design	Population/Setting	*S. aureus*/SAB Relevance	Oral/Dental Relevance	Contribution to Source Identification	Clinical Consequences/Outcomes	Relevance
**Thoresen et al. 2022 [44]**	Norway	Retrospective study	208 adult patients admitted with infective endocarditis who underwent oral infectious focus screening	IE pathogens included viridans streptococci and *S. aureus*; the study compared IE caused by oral bacteria with bacteria of non-oral origin.	All included patients underwent oral focus screening, including clinical/radiologic assessment.	No statistically significant association was found between signs of oral infection and IE caused by viridans streptococci; the authors questioned whether the diagnostic benefit of oral infection screening in IE may be overestimated.	30-day mortality was 4.3%; mortality did not differ significantly between IE caused by viridans streptococci and *S. aureus*.	**Moderate**
**Ostovar et al. 2025 [45]**	Germany	Registry-based observational study with propensity score analysis	530 patients in the Brandenburg Endocarditis Registry who developed IE requiring valve surgery	*S. aureus* was analyzed among causative organisms; *S. aureus* appeared more frequently in control patients than in those with recent dental treatment, although not significantly.	Two dental exposure groups were analyzed: recent dental treatment and desolate dental status.	The study supports an association between dental treatment or poor dental status and IE, particularly with streptococcal organisms; it is less direct for *S. aureus*-specific source identification.	Early mortality was reported for dental treatment and desolate dental status groups; the authors emphasized prevention strategies in patients undergoing dental treatment or with poor dental care.	**Moderate**
**Ismail et al. 2024 [46]**	United Kingdom	Observational cross-sectional study	392 medical records of patients diagnosed with infective endocarditis	Staphylococcal infections were the most common culprit microorganisms, responsible for 130 hospitalizations; *S. aureus* was associated with factors such as stroke, smoking, intravenous drug use, and dialysis.	The study cross-referenced IE bacteremia organisms with the expanded Human Oral Microbiome Database.	Provides microbiological context by demonstrating overlap between IE-associated organisms and oral microbiome taxa; it does not establish a patient-level dental source.	Culprit microorganisms were associated with IE anatomy and treatment approaches; the authors called for enhanced dental-medical collaboration.	**Moderate**

Author/Year	Country	Study Design	Population/Setting	*S. aureus*/SAB Relevance	Oral/Dental Relevance	Contribution to Source Identification	Clinical Consequences/Outcomes	Relevance
**Kumar et al. 2024 [50]**	USA	Retrospective pediatric hospital cohort	67 children meeting IE inclusion criteria at a pediatric hospital	One-third of cases involved *S. aureus* and viridans streptococci; *S. aureus* is discussed as an increasingly important pediatric IE pathogen.	Dental history and dental consultation during admission were assessed; 24 patients had dental consultation.	Provides context on the role of dentists and dental consultation in pediatric IE, but does not prove dental origin of *S. aureus* IE.	Mean hospitalization was 25 days; mortality was 9%; 61% required surgery.	**Moderate**
**Delahaye et al. 1995 [47]**	France	Epidemiologic survey	386 cases of definite, probable, or possible IE in three French regions, 1990–1991	Staphylococci accounted for 20.6% of cases, with *S. aureus* representing more than three quarters of staphylococcal cases.	A dental portal of entry was identified or presumed in 26.0% of cases.	Provides early epidemiologic evidence that dental portals of entry are commonly considered in IE; not specific to SAB source identification.	12.2% died within the first month; 16.1% died within two months.	**Moderate to low**
**Loupa et al. 2004 [48]**	Greece	Prospective observational study	101 IE cases in a tertiary hospital and heart surgery center in Athens	*S. aureus* was the most important pathogen, isolated in 22% of cases; six of 22 *S. aureus* isolates were methicillin-resistant.	Thirteen patients had undergone dental procedures within the relevant prediagnostic period.	Supports dental procedures as one of several antecedent exposures in IE, while *S. aureus* was prominent overall; source attribution remains nonspecific.	Surgical intervention was performed in 51.5%; overall mortality was 16%.	**Moderate to low**
**Nakatani et al. 2003 [49]**	Japan	Nationwide questionnaire survey	848 IE cases from 277 hospitals in Japan, 2000–2001	Gram-positive cocci predominated, including 221 staphylococci; MRSA was found in 7.3% of cases.	Post-dental procedures were the second most common identifiable etiology after unknown origin.	Provides epidemiologic context linking dental procedures with IE etiology, but does not specifically address dental source identification in SAB.	Used to inform Japanese IE prevention and management guidelines.	**Moderate to low**

Extraction Table – Oral Reservoir and Microbiological Evidence

Author/Year	Country	Study Design	Population/Setting	*S. aureus*/SAB Relevance	Oral/Dental Relevance	Contribution to Source Identification	Clinical Consequences/Outcomes	Relevance
**Smith et al. 2013 [24]**	United Kingdom	In vitro biofilm study using clinical MRSA isolates	28 clinical MRSA biofilm isolates from oral and bloodstream sources	Focused on MRSA biofilm behavior rather than SAB as a clinical syndrome.	Evaluated oral MRSA biofilms and the effect of	Supports the concept that MRSA may persist in the oral cavity as a biofilm reservoir; however, it	None of the tested mouthwashes completely eradicated MRSA biofilms; the authors highlighted	**Moderate**

Author/Year	Country	Study Design	Population/Setting	*S. aureus*/SAB Relevance	Oral/Dental Relevance	Contribution to Source Identification	Clinical Consequences/Outcomes	Relevance
					commercial mouthwashes.	does not evaluate dental source identification in SAB.	potential infection-control implications.	
**Koukos et al. 2015 [25]**	Greece	Cross-sectional microbiological study	154 systemically healthy participants with periodontal health, gingivitis, or chronic periodontitis	Investigated oral *S. aureus* and mecA as a marker of MRSA; not a SAB study.	Plaque and tongue samples were tested for *S. aureus* and MRSA.	Demonstrates that *S. aureus* can be detected in the oral environment, although the authors interpreted it mainly as transient flora and found no MRSA.	*S. aureus* was detected in 18% of participants; mecA was not detected in any *S. aureus* isolate.	**Moderate**
**Kwapisz et al. 2020 [26]**	Poland	Molecular epidemiologic study	139 oral *S. aureus* isolates obtained from 2,327 oral samples from dental patients	Examined MSSA/MRSA, virulence genes, resistance profiles, and clonal distribution; not a clinical SAB cohort.	Directly investigated oral *S. aureus* strains from dental patients.	Strongly supports the oral cavity as a potential reservoir of toxigenic and resistant *S. aureus* strains that may disseminate to other body sites.	Twelve isolates were MRSA; SCCmec IV predominated among MRSA strains; virulence genes including enterotoxin genes and PVL-positive CA-MRSA strains were identified.	**Moderate to high**
**Vellappally et al. 2017 [27]**	Saudi Arabia / India	Microbiological antimicrobial-resistance study	*S. aureus* strains isolated from dental caries specimens	Focused on MRSA and vancomycin-resistant *S. aureus* rather than clinical SAB.	Dental caries specimens were used as the oral source material.	Supports the possibility that carious lesions may harbor multidrug-resistant *S. aureus* strains, but does not establish bloodstream spread or source attribution.	Among 150 tested strains, 98 were MRSA; vancomycin resistance markers were assessed, including vanA and vanB.	**Moderate**

Extraction Table – Procedure-Associated Bacteremia and High-Risk Host Evidence

Author/Year	Country	Study Design	Population/Setting	*S. aureus*/SAB Relevance	Oral/Dental Relevance	Contribution to Source Identification	Clinical Consequences/Outcomes	Relevance
**Adeyemo et al. 2013 [29]**	Nigeria	Prospective clinical study	90 patients undergoing cleft lip and/or palate surgery	Coagulase-positive *S. aureus* was isolated from postoperative blood cultures; however, this was transient procedure-associated bacteremia rather than clinical SAB.	Oral and maxillofacial surgical setting; cleft lip repair, cleft palate repair, and alveoloplasty.	Demonstrates that oral/maxillofacial surgery can produce polymicrobial bacteremia including *S. aureus*, but does not address diagnostic source	Bacteremia occurred in 38.1% of cases and persisted for at least 15 minutes in a subset; findings were discussed in relation to prophylaxis for at-risk patients.	**Low to moderate**

Author/Year	Country	Study Design	Population/Setting	*S. aureus*/SAB Relevance	Oral/Dental Relevance	Contribution to Source Identification	Clinical Consequences/Outcomes	Relevance
						identification in established SAB.		
**Akbulut et al. 2018 [30]**	Turkey	Prospective clinical study	28 systemically healthy orthodontic patients undergoing debonding after fixed orthodontic treatment	*S. aureus* was detected in blood cultures after cleaning composite residues and plaque deposits, but not in the context of clinical SAB.	Orthodontic debonding and plaque/composite removal.	Shows that dental/orthodontic manipulation may cause transient bacteremia including *S. aureus*; the study is relevant to biological plausibility rather than SAB source workup.	Bacteremia was detected in 36% of post-cleaning blood samples; authors suggested that prophylaxis may be considered for high-risk patients.	**Low to moderate**
**Akintoye et al. 2002 [31]**	USA	Retrospective cohort study	77 patients undergoing myeloablative allogeneic HSCT after pretransplant dental evaluation	*S. aureus* was among the common organisms recovered from positive blood cultures within 100 days after HSCT.	Periodontal status was assessed radiographically before HSCT.	Evaluated whether advanced periodontal disease was associated with septicemia of likely periodontal or oral origin; no significant association was found.	63.6% had septicemia-associated positive blood cultures; no relationship was found between radiographic periodontal status and septicemia or mortality.	**Moderate**
**Mawardi et al. 2016 [32]**	USA	Retrospective case series	11 patients with multiple myeloma and MRONJ undergoing hematopoietic cell transplantation	*S. aureus* bacteremia occurred in 3 patients; however, no patient developed infection or worsening symptoms at the MRONJ site during hospitalization.	Medication-related osteonecrosis of the jaw in immunocompromised patients undergoing HCT.	Important negative/tempering evidence: MRONJ was present, but the jaw lesion was not clinically implicated as the source of *S. aureus* bacteremia.	Four patients developed fever; median hospital stay was 17 days; MRONJ was not worsened by HCT.	**Moderate**
**Egwari et al. 2008 [33]**	Nigeria	Prospective study	12 patients with postsurgical sepsis after surgery for odontogenic tumors	*S. aureus* accounted for 16.1% of aerobic isolates; infections were polymicrobial.	Odontogenic tumors and postsurgical oral/maxillofacial sepsis.	Demonstrates that postoperative sepsis after odontogenic tumor surgery may involve *S. aureus*, but it does not evaluate SAB or dental source identification.	Noncompliance and inadequate follow-up were linked to earlier development of sepsis; antibiotic therapy was guided by susceptibility testing.	**Moderate**
**Constantinescu et al. 2018 [34]**	Romania	Microbiological resistance study	9,036 bacterial strains from hospitalized patients, including cases with oro-maxillofacial portal of entry	Focused on antimicrobial resistance in *S. aureus* and other pathogens implicated in sepsis; not a clinical SAB cohort.	Oro-dental/oro-maxillofacial portal of entry was explicitly addressed.	Supports the concept that oro-maxillofacial infections may contribute to systemic sepsis and that *S. aureus* resistance	High resistance rates were reported for *S. aureus*, including 94.7% resistance to penicillin; vancomycin remained active in tested strains.	**Moderate**

Author/Year	Country	Study Design	Population/Setting	*S. aureus*/SAB Relevance	Oral/Dental Relevance	Contribution to Source Identification	Clinical Consequences/Outcomes	Relevance
						patterns matter for treatment.		
**Olczak-Kowalczyk et al. 2012 [35]**	Poland	Clinical microbiological study	45 pediatric patients, including organ transplant recipients and children receiving antitumor chemotherapy	*S. aureus* was associated with oral lesions in patients without secondary immunodeficiency; bacteremia accompanied oral lesions in 30% of immunocompromised patients.	Oral mucosal lesions, mucositis, stomatitis, and damaged mucosal barrier.	Suggests that damaged oral mucosa may be associated with bacteremia in immunocompromised children, but does not specifically establish *S. aureus* as a bloodstream source.	Blood cultures were performed in immunocompromised patients with systemic infection; oral lesions were accompanied by bacteremia in a subset.	**Moderate**
**Peterson et al. 1987 [36]**	USA	Clinical microbiological study	24 myelosuppressed cancer patients with 27 acute exacerbations of preexisting periodontal disease	*S. aureus* was among predominant organisms in acute periodontal infections; concomitant bacteremias occurred in two patients, although not specifically *S. aureus* bacteremia.	Acute periodontal infection during chemotherapy-induced myelosuppression.	Suggests that the periodontium may represent a portal of entry for bacteremia during reduced host defenses.	Acute periodontal infections developed mainly at low granulocyte levels; pathogens typical of infections in myelosuppressed patients were recovered from periodontal sites.	**Moderate**
